# Chronic gut inflammation impairs contextual control of fear

**DOI:** 10.1038/s41598-022-24901-3

**Published:** 2022-11-29

**Authors:** C. E. Matisz, M. Patel, N. S. Hong, R. J. McDonald, A. J. Gruber

**Affiliations:** University of Lethbridge, Canadian Centre for Behavioral Neuroscience, 4401 University Drive, W, Lethbridge, AB T1K 3M4 Canada

**Keywords:** Neuroscience, Diseases, Gastroenterology

## Abstract

Chronic inflammatory diseases are highly comorbid with anxiety in humans. The extent to which chronic inflammation is responsible for this relationship remains to be determined. We therefore tested the hypothesis that prolonged, but not brief, gut inflammation is sufficient to evoke anxiety-related behaviours in mice. We used the discriminative fear to context conditioning paradigm to assess fear generalization, which is a prominent feature of anxiety disorders. Gut inflammation was induced by exposure to dextran sodium sulfate (DSS) in the drinking water, a well-established rodent model of ulcerative colitis evoking prolonged inflammation. Neither acute (1 × 5 day cycle) nor chronic (3 × 5 day cycles) exposure to DSS affected fear responses when tested shortly after conditioning. Mice in all groups generated more fear responses (freezing) in a chamber previously paired with mild shock, as compared to a chamber with no pairing. This suggests DSS exposure had no effect on acquisition or expression of conditioned fear. Acute and control animals showed this same contextual control of freezing when tested 9 days later. In contrast, at this remote time point, the chronically treated animals exhibited increased freezing in the unpaired chamber such that freezing was equivalent in both contexts. These animals, however, showed intact preference for the unpaired chamber when allowed to freely move between chambers. These data suggest that some mnemonic process engaged after training, such as memory consolidation, is affected by past chronic inflammation so as to generalize negative associations and engage fearful responding in inappropriate contexts, despite intact knowledge that the chambers have different affective associations sufficient for place preference.

## Introduction

Most peripheral chronic inflammatory diseases, such as rheumatoid arthritis, diabetes, and cardiovascular disease, are comorbid with mood disorders^1–3^. This is especially evident in inflammatory bowel diseases (IBD). An estimated 50% of patients with IBD are diagnosed with anxiety disorder^4^, which persists even during periods of disease remission. A primary feature of anxiety disorder is increased expectation of negative affect states, typically experienced as fear or dread, and the activation of associated responses such as engagement of the sympathetic system^5^. The generalization of these states and responses to unconditioned (e.g. safe) contexts and stimuli is a fundamental feature of anxiety disorders^6,7^, and likely contributes to the etiology and maintenance of such disorders^8^.

Although the psychological burden of disease likely contributes to anxiety in IBD, overwhelming clinical evidence indicates that gut-induced neuroinflammation is a central mechanism^9^. This is further supported by studies of gut inflammation in animals, which also typically report increased anxiety-like behaviours^9^. To date, most of the animal work has focused on acute gut inflammation, which does not recapitulate the chronic and relapsing nature of inflammation in IBD. Moreover, these prior studies predominantly use tests of anxiety, such as elevated plus maze and open field tasks, which lack the learning and contextual component essential for assessing affect-related associations involved in fear generalization.

Context refers to a configuration of environmental features. Rodents and other animals can rapidly associate specific contexts with negative affective outcomes. Delivering a stimulus that evokes negative affect (e.g. footshock) in only one of two distinct contexts typically evokes greater fear-related behaviours (e.g. freezing) in the context previously paired with the stimulus as compared to the unpaired one. This is called discriminative contextual fear conditioning. Few previous studies have tested the effects of peripheral inflammation on contextual fear. One of these studies found that acute peripheral inflammation evoked by a single injection of the immunogenic bacterial antigen lipopolysaccharide (LPS) was associated with impaired discriminative fear conditioning to context when tested 6 h later^10^. This suggests that inflammation or its after-effects can modulate discriminative fear, but it remains unknown if this effect persists over many days.

Mammals are highly adept at using past experience to guide responses, and can use generalization as a strategy to guide behavioural output in novel situations. Generalization occurs when a conditioned response is induced by a novel stimulus or context with some resemblance to the conditioned stimulus/context^11,12^. This often has adaptive value, but can be maladaptive if responses become over-generalized^8,13^. The excessive fearful behaviour observed in anxiety disorders has been proposed to be a consequence of fear overgeneralization^6^. This suggests that discriminative fear conditioning to context (DFCTC) is a useful tool to measure neural processing as an indirect metric of anxiety. Specifically, an increase in fear-related behaviors in the unpaired context suggests overgeneralization of fear consistent with neural processing changes proposed to be involved in anxiety disorders. We therefore hypothesize that long-lasting gut inflammation should disrupt contextual fear conditioning in rodents. We tested this hypothesis in animals receiving an acute (1 × 5 day) or chronic (3 × 5 day) course of gut inflammation.

## Results

**Figure 1:**
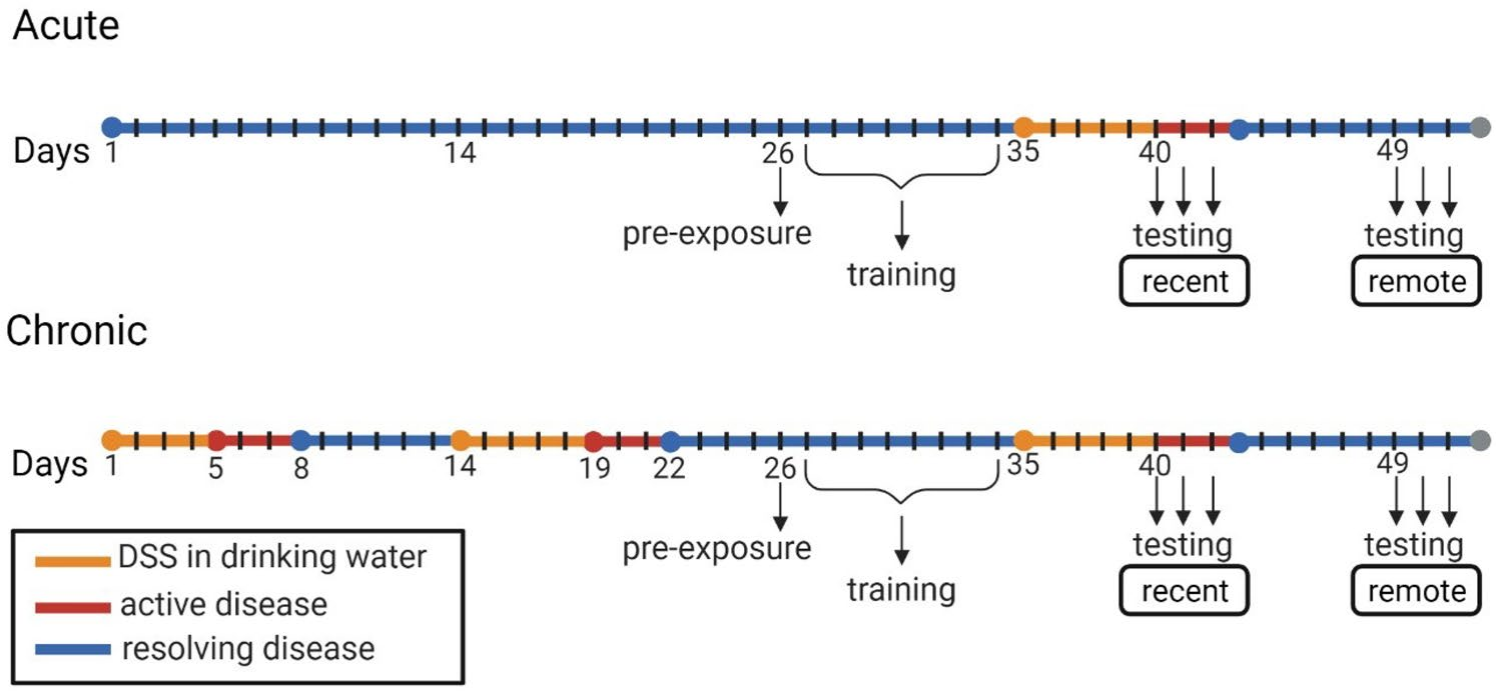
Timeline of experimental procedure. One cycle of DSS consists of 2.75–3% wt/v DSS in the drinking water for 5 days (orange line), followed by regular drinking water. The severity of disease activity peaks 6–8 days post exposure to DSS within a cycle (indicated by red line), at which point some clinical signs of disease begin to resolve (in blue). Mice assigned to the chronic DSS treatment receive two cycles of DSS prior to pre-exposure (day 26) and training (days 27–34) in the fear to context paradigm. On day 35, chronic DSS mice receive their third cycle of DSS, and acute DSS mice receive their first cycle of DSS. Controls receive regular drinking water throughout the experiment. Fear to context testing and place preference testing occur during recent (days 40–42) and remote intervals (days 49–51). N = 12 per treatment group.

**Figure 2:**
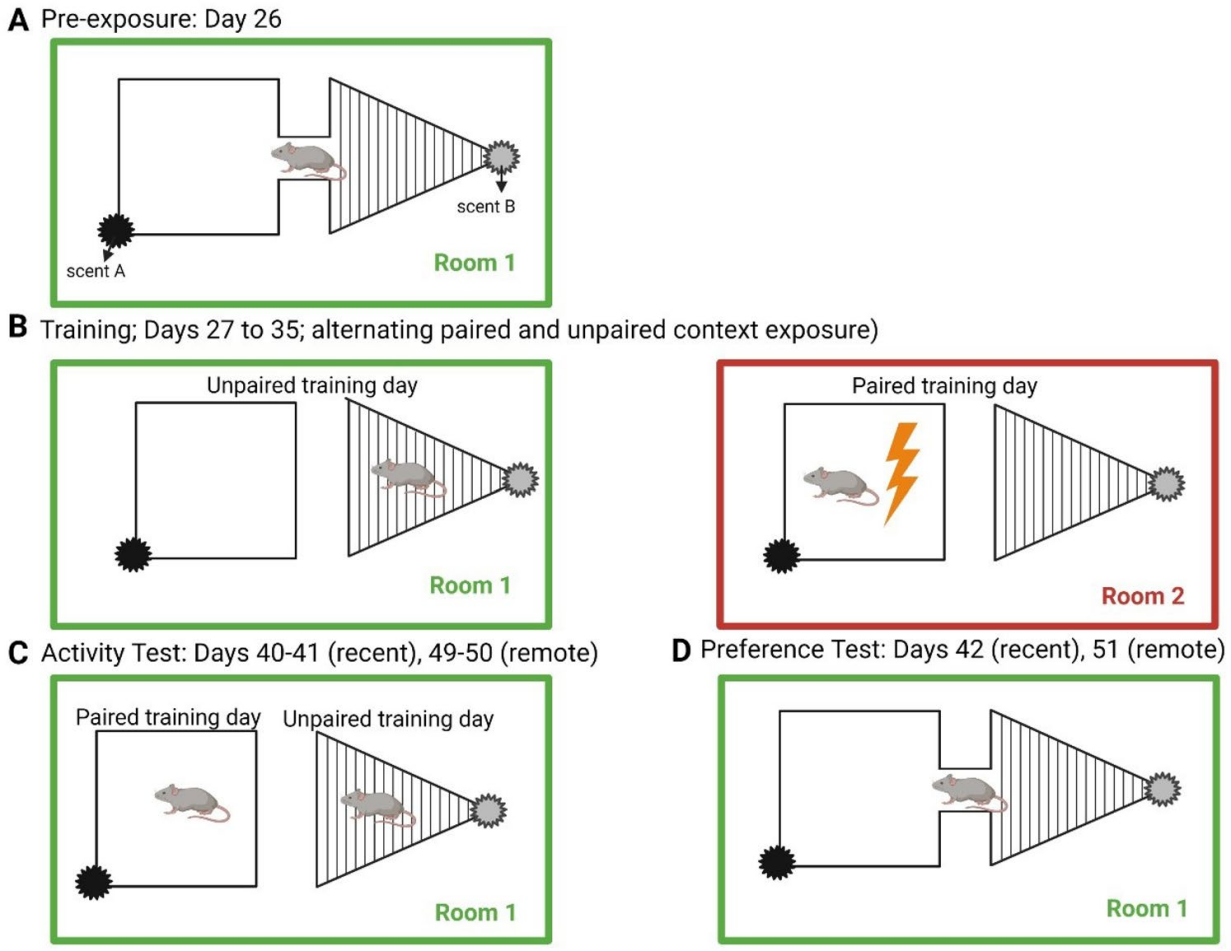
Schematic representation of the discriminative fear to context apparatus during training and testing. Two chambers differ in shape (rectangle, triangle), colour (white, black stripes), and odor (cinnamoaldehyde, gerenyl-formate), respectively. The scented gauze is placed at the top of the chambers, and held in place by a Plexiglas lid. (**A**) Pre-exposure: animals freely explore both chambers, connected by a Plexiglas hallway, for 10 min. (**B**) Training: paired and unpaired context training occur on alternate days (in separate rooms), for 5 min each day over 8 days. (**C**) Fear testing; freezing behaviour was recorded in the paired and unpaired context on separate days in the safe room (room 1). (**D**) Preference test; dwell time in each context was recorded (testing conducted in room 1).

Dextran sodium sulfate (DSS) added to the drinking water of mice is a well-validated model used to evoke inflammation in the colon, the severity of which can be assessed via weight loss and disease activity score^14^. In the present study, treatment groups included an acute exposure to DSS (one 5-day cycle), a chronic exposure to DSS (three 5-day cycles), and negative controls (untreated water). Mice assigned to the acute treatment group were trained on the DFCTC task prior to their first cycle of DSS, while mice in the chronic treatment group were trained between their 2nd and 3rd cycle of DSS (Fig. 1). Testing for DFCTC occurred across two separate rooms, at recent and remote time intervals, using two chambers with distinct contexts, as illustrated in Fig. 2.

### Exposure to DSS evoked elevated markers of gut inflammation

**Figure 3:**
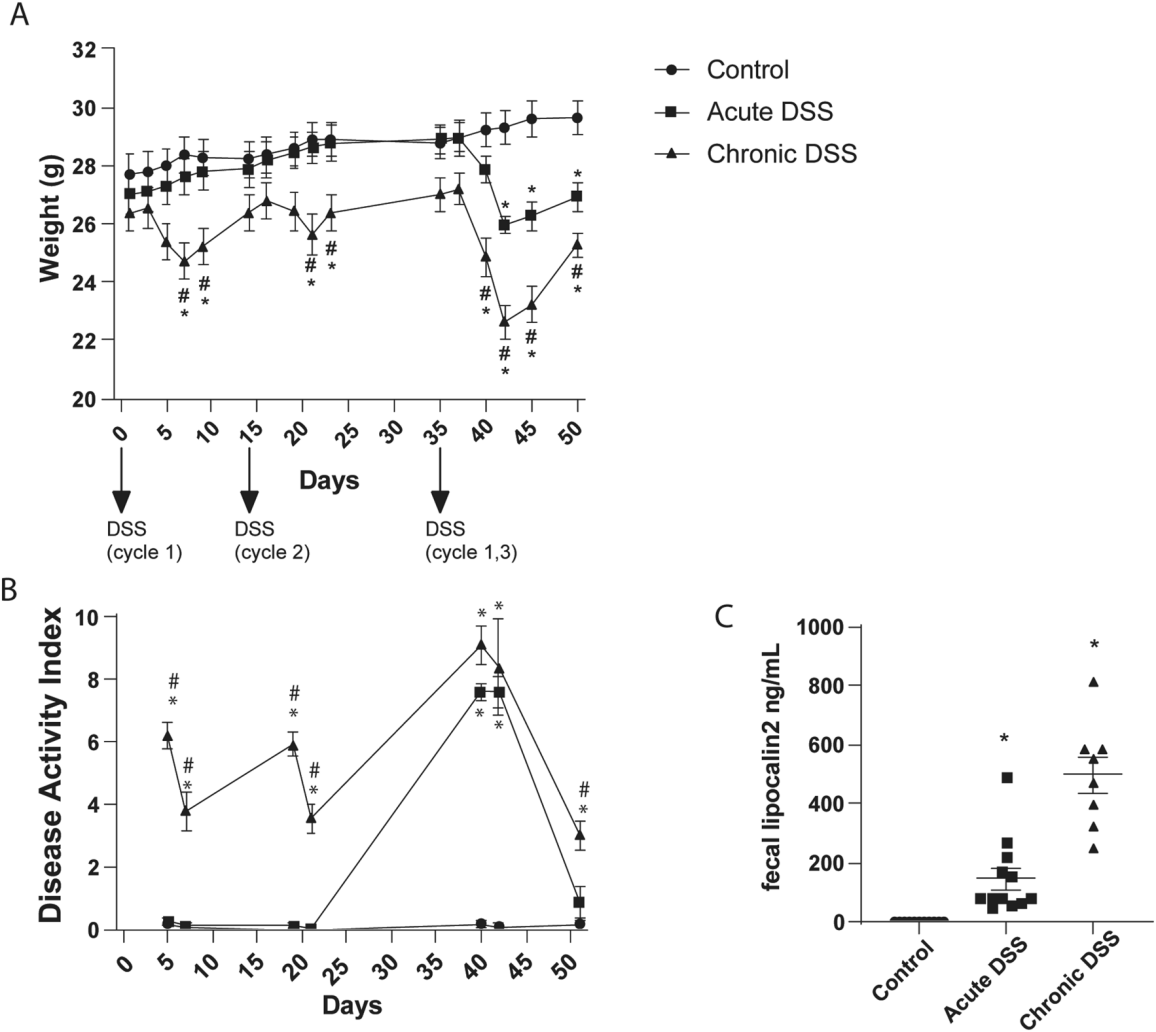
Indicators of gastrointestinal dysfunction and inflammation. (**A**) Body weight and (**B**) Disease activity index, and (**C**) fecal marker of GI inflammation (lipocalin-2 levels on day 51) among treatment groups. Data represents the mean ± SEM, and are the aggregate from fully counterbalanced cohorts run in two separate experiments (n = 11–12 per group). Asterisks ‘*’ indicates p < 0.05 versus control, and hash ‘#’ indicates p < 0.05 versus acute DSS, as determined by two-way RM ANOVA with Tukey’s post-test (**A**), mixed model analysis with Tukey’s post-test (**B**), and Brown-Forsythe ANOVA (**C**), with Dunnett’s post-test.

Mice exposed to DSS in the present study had reduced body weight (Fig. 3A) relative to controls, both during the DSS treatment (Two-way RM ANOVA; F_2,31_ = 8.499, p = 0.0011) and 8 days after treatment cessation (F_2.298,71.24_ = 14.35, p < 0.0001). Likewise, the disease activity score was increased by DSS treatment (Fig. 3B, Mixed model analysis, F_2,32_ = 139.6; p < 0.0001) and time (F_2.474,65.96_ = 78.85; p < 0.0001). Lipocalin-2, a well-validated biomarker of gut inflammation^15^, was measured in fecal samples collected on the final day of the study (day 51). Exposure to DSS was associated with elevated levels of fecal lipocalin-2 relative to controls (Fig. 3C, Brown-Forsythe ANOVA, F_2,12.65_ = 34.82, p < 0.0001; Dunnett’s post-hoc test: acute, p = 0.0058; chronic, p = 0.0003). Mice chronically exposed to DSS exhibited greater weight loss, disease activity scores, and elevated fecal lipocalin-2 compared to mice with an acute exposure to DSS, suggesting a cumulative effect of inflammation over the course of administration.

### Gut inflammation did not affect contextual fear responses one week after training

**Figure 4:**
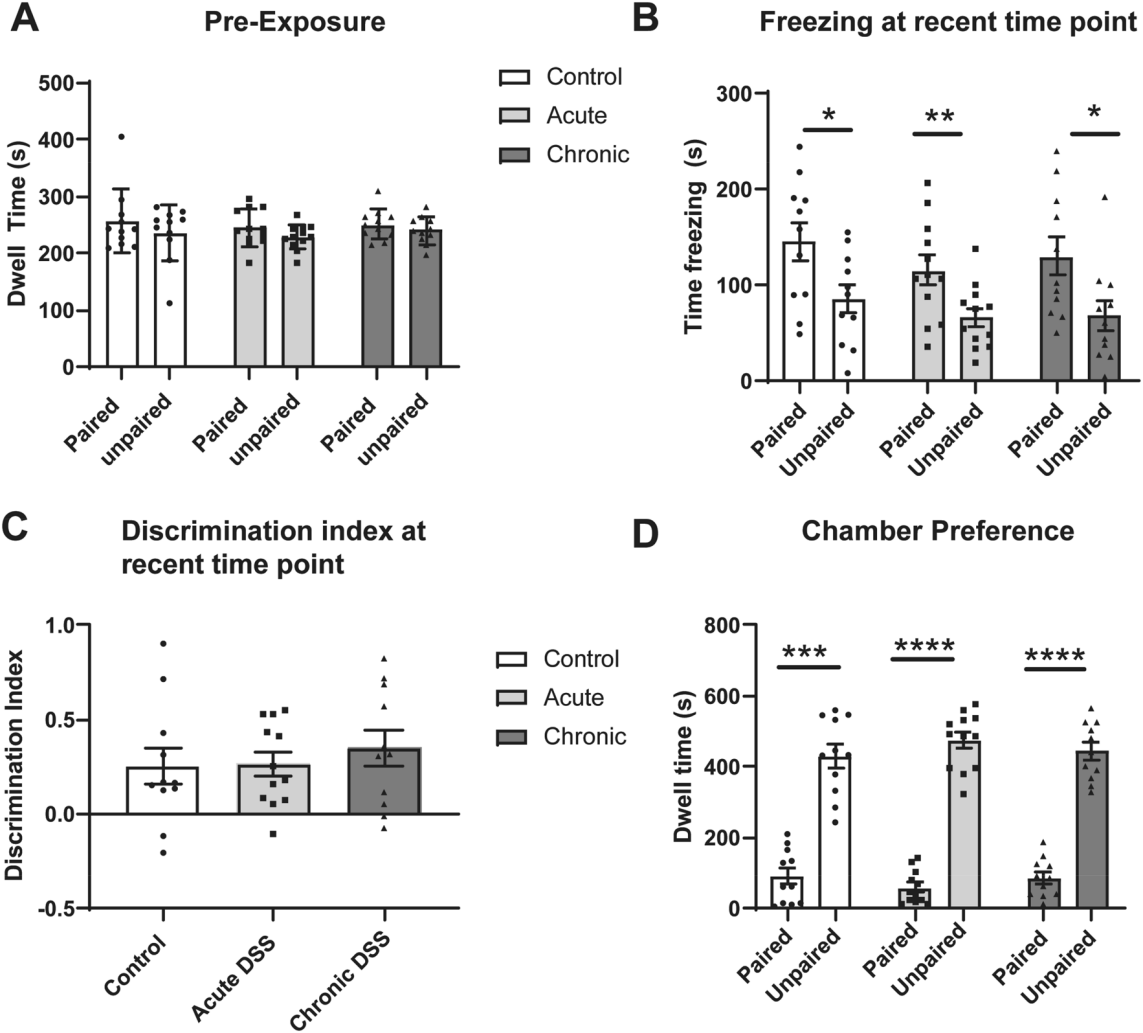
Freezing behaviour and chamber preferences at the recent time interval 6–7 days after training. (**A**) Total time spent in chambers when allowed to freely explore the apparatus, prior to fear training. (**B**) Total time freezing in paired and unpaired contexts during discriminative fear to context testing at the recent test interval. (**C**) Discrimination index of freezing behaviour, computed as the relative fraction of time freezing in the paired context versus the unpaired context. (**D**) Dwell time in paired and unpaired chambers. *p < 0.05; **p < 0.01; ***p < 0.001. ****p < 0.0001 computd by mixed effects repeated measures ANOVA and Sidak post-test (**A**,**B**,**D**), or one-way ANOVA and Tukey’s post-test. Data are mean ± SEM (n = 11–12).

The DFCTC task involves pairing one of two chambers with a mild shock, and the other with no shock (Fig. 2). The behavioural readouts are (i) the time spent freezing (a species-specific sign of fear in rodents) in each chamber, and (ii) the time spent in each chamber when animals have free access to both. It is important to assess any intrinsic preference of each mouse for either chamber prior to training, which could confound the results. Mice were therefore allowed to freely locomote between chambers via a connecting hallway prior to conditioning. We assigned mice that spent more time in one chamber to the group that will receive shock in that preferred chamber. Mice within each group were further assigned into subgroups to control for the order in which they would be trained and tested (e.g. paired on day 1 vs unpaired on day 1). Mice were trained prior to their first cycle of DSS exposure (acute DSS group) or between the 2nd and 3rd cycle of DSS exposure (chronic DSS) (Fig. 2). Post assignment statistical testing revealed no significant effects on chamber preference by group (p = 0.3184), context (p = 0.1934), or group-context interaction (p = 0.9445) (Fig. 4A). We tested their responses at two time points: at a recent time (6–7 days after training completion); and a remote time (17–18 days after training completion). Freezing responses are tested in only one context per day to avoid cross-over effects, thus requiring two days. During the recent time point, mice in all groups spent more time freezing in the paired context relative to the unpaired context [Fig. 4B; F_1,11_ = 33.89, p = 0.001; Sidak post-test, control (p = 0.0436), acute (p = 0.0080), and chronic (p = 0.0161)]. No significant differences in the discrimination index (difference in freezing between contexts) was observed between treatment groups (Fig. 4C; F_2,31_ = 0.3807, p = 0.6865). These data indicate that neither acute nor chronic DSS treatment affected memory acquisition, recall, or freezing responses.

The day following the two testing days, animals were allowed to locomote freely between both chambers via a Plexiglas connecting hallway. This allows us to assess the animals’ preference for either chamber, as an indicator of threat assessment independent of freezing. Animals in all groups had greater dwell time in the unpaired context relative to the paired context (Fig. 4D; F_1,11_ = 326.3, p =  < 0.0001. Sidak post-test; control (p = 0.0004), acute (p < 0.0001) and chronic (p < 0.0001). This further suggests that DSS did not affect the ability of mice to encode or use information about asymmetric valuation among chambers.

In sum, we found no effects of either acute or chronic DSS treatment on contextual fear discrimination or place preference one week after training.

### Chronic DSS impaired contextual control of freezing 17 days after training

**Figure 5:**
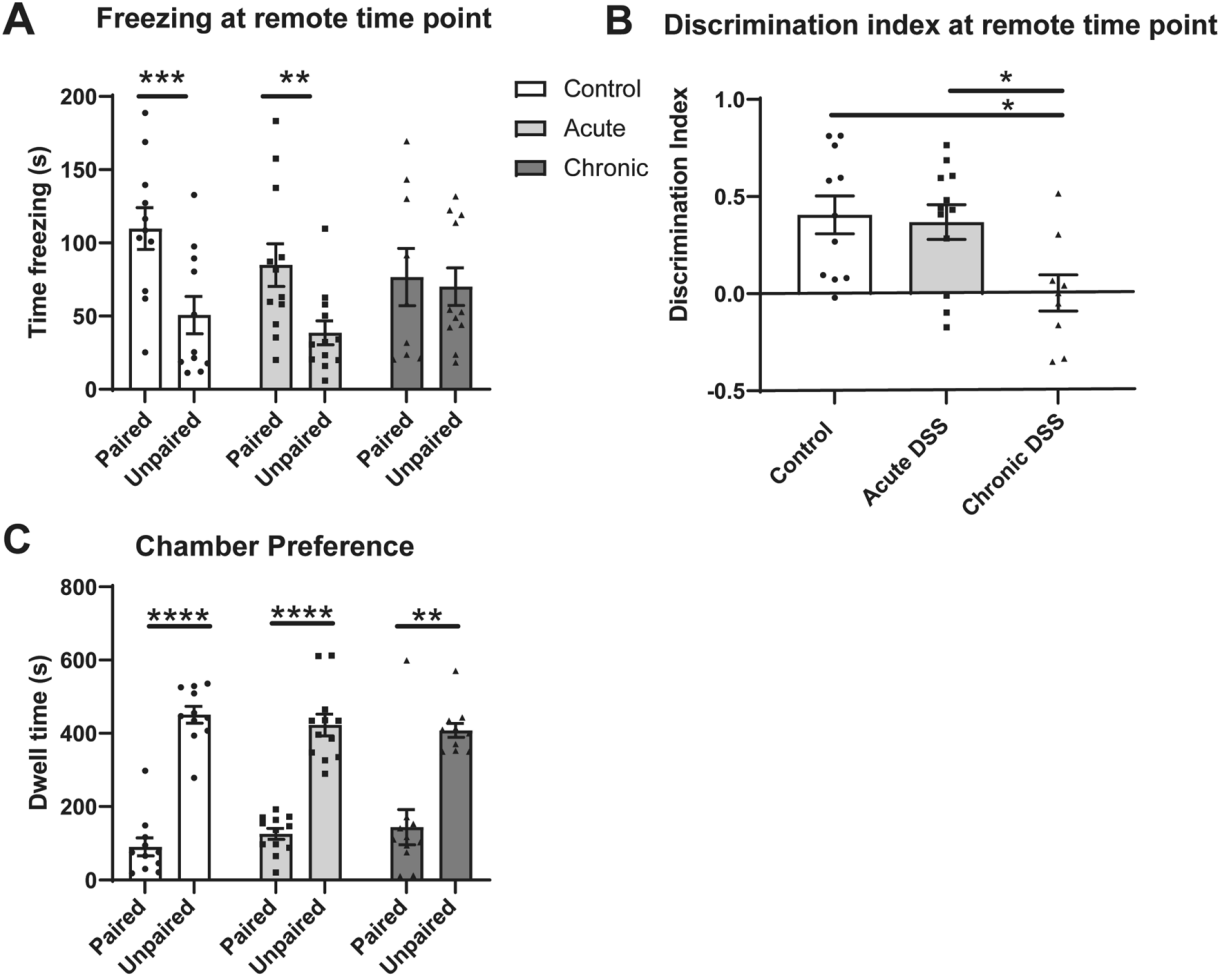
Freezing behaviour and chamber preferences at the remote testing interval (17–18 days after training). (**A**) Total time freezing in paired and unpaired contexts during discriminative fear to context testing at remote time point. (**B**) Discrimination index of freezing behaviour; Time spent freezing in unpaired-paired context, divided by the total freezing time at remote time point. (**C**) Dwell time in paired and unpaired chambers Mixed effects repeated measures ANOVA with Sidak post-test; *p < 0.05; **p < 0.01; ***p < 0.001; ****p < 0.0001 computed by mixed effects repeated measures ANOVA and Sidak post-test (**A**,**C**), or one-way ANOVA and Tukey’s post-test (**B**). Data are mean ± SEM, n = 9–12.

Animals were re-tested starting 9 days after completion of the first test. Mice in the control or acute DSS groups again froze significantly more in the paired than unpaired context [Fig. 5A; F_1,29_ = 20.28; control (p = 0.00098), acute DSS (p = 0.0065)]. Mice with previous chronic DSS exposure, however, froze equally in the paired and unpaired context (p = 0.9373). The discrimination index of chronic DSS-treated mice was significantly lower than the control (p = 0.0178), and acute DSS (p = 0.030) groups (Fig. 5B, F_2,29_ = 5.088, p = 0.0128). These data indicate that only the mice treated with chronic DSS showed a loss of discriminative fear responses among the contexts. The amount of freezing in the paired chamber was not different from the other groups (F_2,29_ = 1.157, p = 0.3286). Rather, they engaged in more freezing in the unpaired context. This suggests that mice with previous chronic exposure to DSS generalized fear to the unpaired context during the remote test interval.

Even though mice chronically treated with DSS froze equally in both chambers, their preference for the unpaired chamber was not different than for the other groups (Fig. 5C; F_1,11_ = 175.3, p < 0.0001. Sidak post-test; control (p < 0.0001), acute (p < 0.0001) and chronic (p = 0.0041)). Thus, chronic DSS treatment appears to have affected learning and memory systems involved in the control of freezing behaviour, but not the systems involved in guiding place preference during the remote test interval.

## Discussion

These data reveal three important findings. First, neither acute nor chronic gut inflammation impairs the acquisition or short-term expression of contextual fear. That is, regardless of treatment group, all animals were able to learn context-specific fear during training and recall this information when tested at the recent time point. This result suggests that the neural machinery and plasticity mechanisms necessary for learning and remembering what context is predictive of a fearful event was functional. Second, only mice with chronic gut inflammation fail to exhibit context-specific fear behaviour when tested during the remote test interval 9 days later. This suggests that the chronic DSS treatment affected brain processes occurring between the recent and remote testing, such as neural hypoactivity or memory consolidation^16^. Third, the learning and memory systems involved in context preference were not impacted by either acute/chronic DSS at either recent/remote testing points. This result suggests that there is some difference in the cognitive functions and brain networks necessary to control fear responses when forced to be in the context, compared to when the subject can simply avoid them. In sum, chronic gut inflammation appears to have altered the function of neural systems involved in threat responding, despite intact knowledge of asymmetric risk/value needed for place preference.

### What brain regions may be affected to produce the observed fear generalization?

The published literature on contextual fear memory affords some inference regarding the neural structures likely contributing to the fear generalization observed in the present experiment. There is general consensus from lesion, retrograde tracing, and behavioural pharmacology studies that contextual fear memory requires coordinated activity of the hippocampus (HPC), dorsomedial prefrontal cortex (dmPFC), and amygdala^17–22^. Neurons in the HPC encode contextual representations, which can be integrated with aversive signals in the basolateral amygdala (BLA) to produce a fear memory (for review, see Ref.^23^). Projections from the BLA to the central nucleus of the amygdala (CeA) initiate fear and defensive responses via multiple projections. These include the periaqueductal gray-cerebellar circuit^24^, which engages freezing responses^25^ and the hypothalamus–pituitary–adrenal axis, which activates sympathetic responses such as increased respiration rate^26^. Inputs from the dmPFC are probably less involved in acquiring and/or storing the association during fear conditioning, but might play an important role in selecting response patterns^17,27^. For instance, the infralimbic and prelimbic regions of medial PFC are involved in discriminative fear responses in rodents with lesioned hippocampi^28,29^. Pharmacological lesioning or blocking synaptic transmission of the medial prefrontal cortex (infralimbic, prelimbic, and anterior cingulate) has been shown to impair contextual fear discrimination^30,31^. Furthermore, dmPFC activity shifts when animals transition between fearful and safe contexts, and activation of a population of dmPFC neurons that project to the vlPAG is necessary and sufficient for contextual fear discrimination^32^. This pattern of effects suggests that fear conditioning to context normally involves interactions among dmPFC, amygdala, hippocampus, and various hypothalamic and brainstem regions, but can be accomplished without the contribution of the hippocampus in some instances^33,34^.

The results of the present experiment are unique in that they suggest that chronic gut inflammation affects the brain in a way that alters the ability to control fear responses based on context, but does not impact the ability to learn and remember the context-fear associations. However, it should be noted that there may be other effects of previous exposure to DSS on memory acquisition that are not captured by the fear to context paradigm used in this study. Because neural plasticity in both the amygdala and hippocampus are involved in such context-fear associations^23^, the lack of effect of chronic DSS on responding shortly after training suggests these are unlikely to be the primary sites affected by chronic gut inflammation. Further, the hypothalamic and brainstem regions important for producing various fear responses do not seem to be compromised either, because the rate of fear responses in the paired context was not affected. The likely target is a higher-order brain region, such as the PFC, which is less involved in basic associative learning and memory processes per se, but is important for decision making and response selection^27^. However, because neurotoxic damage spanning multiple regions in the medial prefrontal cortex impairs both discriminative freezing and place preference in the DFCTC^30^, widespread effects of prolonged gut inflammation among its various subregions is unlikely. It is possible, however, that a specific subregion (prelimbic, infralimbic, or anterior cingulate) is impacted, but there is currently no reported data regarding the role of each region in freezing versus preference measures in the DFCTC. Interestingly, rats with neurotoxic damage to the infralimbic/prelimbic regions with spared anterior cingulate showed normal acquisition of the DFCTC task^29^, suggesting the anterior cingulate might be important for fear generalization. Further research is required to assess this idea. Although the orbitofrontal prefrontal cortex (OPFC) has received much less attention in the contextual fear literature, it appears to play a role. Rats with lesions or reversible inactivations of OPFC exhibited strikingly similar deficits as the animals treated with chronic DSS^29,35^. OPFC-lesioned rats exhibited equivalent freezing in both paired and unpaired chambers in the DFCTC paradigm, while still preferring the unpaired chamber in the conditioned preference test.

Taken together, it seems clear that regions of the dmPFC, amygdala, hippocampus, and various hypothalamic and brainstem regions are part of a neural network important for DFCTC. However, it is likely that the impacts of chronic gut inflammation on fear generalization is due to some alteration in prefrontal control of fear responses that is particularly important when the subject is forced to experience those conditioned contextual stimuli with no chance of escape. The limited available evidence suggests that regions of dmPFC do not account for the pattern of results in the present data—specifically, increased freezing in the unpaired context despite a normal preference to dwell there. We therefore speculate that one possible explanation of the present data is hypoactivity of the OPFC following chronic DSS. This does not preclude DSS-related effects on other structures.

### The duration of inflammation impacts memory

The single 5-day cycle of DSS used here impairs short-term memory as assessed by the novel object recognition task^36^. Interestingly, this impairment is not evident in mice given three 5-day cycles, suggesting some tolerization effect^36^. Importantly, this study found no differences in locomotor activity between control, acute DSS, and chronic DSS animals. Although some forms of memory may recover as inflammation continues, the present data suggest that prolonged inflammation has more subtle effects on long-term mnemonic processes in system-specific circuits. Specifically, the emergence of fear generalization 9 days after training. Prior studies have shown increased fear generalization 28–30 days after training^37,38^. A unique feature of the present data is the place preference test, which revealed a normal propensity for animals in all groups to spend more time in the safe context than the one paired with shock. This suggests that the neural systems involved in place preference have intact context representations and affective associations. So it is unlikely that the fear generalization in the animals given repeated DSS is due to a global loss of context discrimination. Moreover, we did not observe fear generalization in the other treatment groups in the present study, even though all groups had equal time between training and testing. This suggests that time alone is insufficient to account for generalization. Research by Correa et al. have revealed an important relationship between cortisol levels and fear generalization over time^39–41^, suggesting neuroendocrine responses influence memory consolidation of discriminative fear behaviours. It is tempting to speculate that chronic gut inflammation accelerates some processes involved in generalization, as part of prolonged inflammation’s purported role in accelerating biological aging^42^. Indeed, studies using systemic LPS injection to model peripheral inflammation^10,43^ suggest neuroimmune processes are likely involved.

## Conclusion

In the present study, animals with chronic gut inflammation showed impaired contextual control of fear responses. They froze more in a safe context not paired with shock, relative to controls and animals with acute gut inflammation. This occurred despite apparently normal learning and acquisition of contextual fear, unaffected rates of freezing in the paired chamber, and intact place preference. We argue that this derives from some alteration of information processing in a higher-order association system, such as the prefrontal cortex. This pattern has been previously observed in rats with lesions of the orbitofrontal region of PFC, suggesting that this structure may be involved in the behavioural phenomena observed here. We speculate that chronic gut inflammation interferes with the ability of OFPC to discriminate contexts (e.g. pattern separation) and/or reduces the relative contribution of OFPC to behavioural control (e.g. it becomes hypoactive). Moreover, this appears to involve memory consolidation or other gradual process because the increased freezing in the safe context only emerged at the later testing time. The single 5-day course of DSS treatment, which produces systemic, self-resolving inflammation, was insufficient to evoke the generalized fear effect. These data reveal that prolonged peripheral inflammation has effects on learning and memory processes involved in context-based regulation of fearful responses, which may help account for the increased risk anxiety following chronic bodily inflammation in animals and humans.

## Materials and methods

### Animals

Male C57Bl/6 mice (8 weeks old, Jackson Laboratories, Bar Harbor, ME) were housed in groups of three in clear cages in the Optimice IVC rack system in a vivarium on a 12:12 light dark cycle, with free access to standard mouse chow and drinking water. Animals were acclimatized to the facility for a minimum of 1 week prior to start of experimentation. A total of 36 mice were used for two separate experimental cohorts (run at different times of the year by different personnel) to ensure reproducibility. All experiments were carried out in accordance with the guidelines of the Canadian Council of Animal Care, were approved by the University of Lethbridge Animal Care Committee, and are in compliance with ARRIVE guidelines.

### Colitis induction and assessment

For the induction of acute DSS, colitis, mice were given 3.25% wt/v DSS (MW 40kD; Fischer Scientific, CAT 9011-18-1) in their drinking water for 5 days, followed by regular drinking water for 9 days. For the induction of chronic DSS colitis, mice were exposed to three cycles of 5-day DSS (2.75%, 2.75–3% 3.25% wt/v). The first cycle was followed by 9 days of regular water, and the second cycle was followed by 16 days of regular water in order to allow time for training on the DFCTC task. The peak of disease within a cycle occurs 6–8 days after cycle initiation, after which the clinical signs of disease begins to resolve. Control mice received regular drinking water for the duration of the experiment (Fig. 1). Body weight and disease activity was monitored throughout the experiment. Disease Activity was based on three measures, and scored as described previously^36^: percentage of body weight lost from the start of the cycle on days 0, 14, and 28, scored on a 1–4 range based on the percentage of body weight lost (0 = 0%, 1 = < 1 to ≤ 5%, 2 = > 5 to ≤ 10%, 3 = > 10 to ≤ 15%, 4 = > 15%); stool consistency (solid fecal pellets = 0, soft, sticky stools = 2, loose, water stools = 4); and the presence of fecal blood (no blood = 0, fecal blood = 4). The total disease score was computed as a weighted average of each of these three measures. Fecal pellets were collected on day 51 of the experiment, and analyzed for levels of lipocalin-2 (Benoit et al.). (see below). Following behavioral experiments (described below), mice were anaesthetized with isoflurane, and euthanized via i.p. injection of sodium pentobarbital (340 mg/kg; Merck & Co, Quebec).

### Quantification of fecal lipocalin-2 by ELISA

Fecal lipocalin-2 is a biomarker for intestinal inflammation in both humans, and rodents. Samples were prepared following previously published protocols. Briefly, frozen fecal samples were reconstituted in PBS with 1% Tween 20 (10 mg/100 µL), and vortexed for 20 min. Samples were centrifuged for 10 min and 12,000 rpm, supernatants collected, and stored at – 20 °C until analysis. Lipocalin-2 levels were quantified in the supernatants using the DuoSet murine Lcn-2 ELISA Kit (R&D Systems, Minneapolis, MN, USA), following the manufacturer’s instructions.

### Fear to context

The experimental design for this study follows Trow et al., with minor modifications^35^. *Apparatus:* Two Plexiglas chambers used in the study included a rectangular chamber (30.3 cm × 26.2 cm × 30.5 cm) with solid-white walls, and an equilateral triangular shaped chamber (26.3 cm × 30.5 cm) with black and white-striped walls. Cinnamoaldehyde and gerenyl-formate were used as the olfactory cues for the square and triangle contexts, respectively. Both chambers included a small door, where a removable Plexiglas hallway connected the two chambers. The flooring of the chambers and the alleyway consisted of parallel metal rods (0.2 cm diameter) placed 0.4 cm apart. The entire apparatus was placed on a clear Plexiglas table, 21 cm from the floor, with an 1080p C922 Logitech Camera placed underneath the table to record animal movements.

#### Pre-exposure

Prior to training, animals were acclimatized to the apparatus to reveal any chamber biases. They were placed in the center of the Plexiglas hallway, and allowed to freely explore the chambers for 10 min. Animals were considered to be occupying a chamber if both forepaws were past the doorway, within the chamber. The dwell time in each chamber was recorded and summated by an observer. A preference to a chamber was defined as an animal that spent significantly more time in one chamber. Animals with a preference for a particular chamber were assigned to receive shock in that preferred chamber, in order to avoid confounds of chamber bias. Further, for each group, half the animals were assigned to receive shock in one chamber, and the other half in the other chamber.

#### Training

The training phase of the experiment began approximately 24 h after pre-exposure to the apparatus. During training, Plexiglas panels were inserted to block access to the connecting hallway. Each animal was placed in one of the chambers for 5 min. In the paired context, mice received a 2 s 0.5 mA footshock delivered at minute 2, 3, and 4. No footshock was delivered in the unpaired context. Paired and unpaired contexts were located in separate ‘shock’ and ‘safe’ rooms, respectively. Animals were exposed to the paired or unpaired context in an alternating fashion for a total of 8 days. The training schedule (order of exposure to context), and the chamber designated the paired context were counterbalanced within and between treatment groups.

#### Activity testing

The testing procedure took place over 2 days in the safe room (Figs. 1, 2). Animals were placed in their assigned context (½ paired, ½ unpaired) and video recorded for 5 min. On the subsequent day, animals were placed in the alternate context, and video recorded for 5 min. The amount of time spent freezing was recorded by an observer blinded to the treatment groups. Freezing was defined as complete immobility of the mouse body, head, and whiskers. Mice were tested during a recent time point of active inflammation (day 40–41), and at a remote time point when disease was resolving (day 49–50).

#### Preference testing

Preference testing took place approximately 24 h after the second day of activity testing, in the safe room, on days 42 and 51. Panels preventing access to the alleyway were removed, and animals were allowed to freely explore both chambers for 10 min. Observers blinded to treatment status of the animals analyzed video recordings to determine the total dwell time spent in each chamber.

### Statistical analysis

Data are presented as mean ± SEM. Statistical significance was set at p < 0.05. Body weight and disease activity was analyzed by a two-way repeated measures ANOVA and repeated mixed effects analysis, respectively, with Tukey’s post-test. Due to significantly different standard deviations among treatment groups, lipocalin-2 expression in fecal samples was analyzed by a Brown-Forsythe ANOVA, with Tukey’s post-test. Dwell time and time freezing within the paired and unpaired context was analyzed by repeated mixed effects model, with a Sidak post-test. The discrimination index was calculated by the fraction of time (expressed as a percentage) spent freezing in the unpaired context, minus fraction of time spent freezing in the paired context, divided by the sum fraction of time spent freezing in both contexts. Statistical analyses were conducted with GraphPad Prism 8.1.2 (GraphPad Software, La Jolla, CA, USA). Figures 1 and 2 were created with biorender.com, and Figs. 3, 4 and 5 were produced using Adobe Illustrator CC 2018.

## Data Availability

The datasets used and/or analyzed during the current study available from the corresponding author upon reasonable request.
